# Supplementary material for: Dedicated photoreceptor pathways in Drosophila larvae mediate navigation by processing either spatial or temporal cues
Source: Nat Commun. 2018 Mar 28;9:1260. doi: 10.1038/s41467-018-03520-5 (PMC5871836; doi:10.1038/s41467-018-03520-5)
Supplement: Supplementary file 1 — Supplementary Information [file 41467_2018_3520_MOESM1_ESM.pdf]

Dedicated photoreceptor pathways in *Drosophila* larvae mediate navigation by processing either spatial or temporal cues

Humberg et al.

Supplementary Information

Supplementary Figure 1

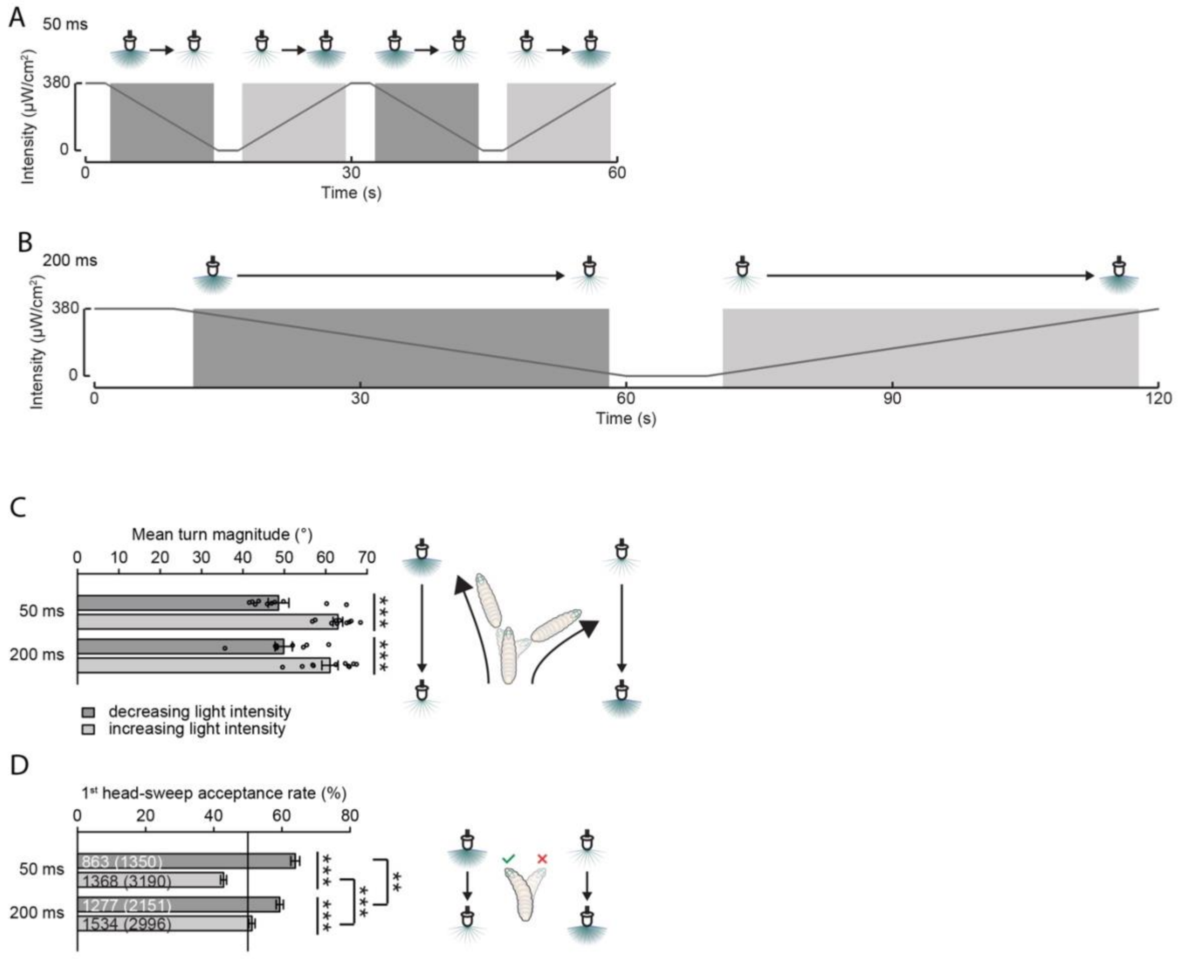

Supplementary Figure 1 Different temporal light gradients

We varied the intensity change speed of the temporal light gradient. (A) We doubled the intensity change speed resulting in a linear light intensity decrease and increase for 12.75 s each. The phases of de- and increasing intensity were spaced by 2.25 s constant high or low light conditions. One cycle lasted 30 s and was repeated until the end of the experiment. (B) We divided the speed of the temporal light gradient by half, resulting in a light intensity de- and increase for 51 s each. These phases were spaced by 9 s constant high or low light respectively. One cycle lasted 2 min and was repeated until the experiment stopped. (A, B) The phases of constant light intensity +/- 1 s were not taken into account for analysis. (C) In both conditions, larvae make larger turns in phases of light intensity increase compared with turns occurring during the phase of light intensity decrease. (D) Both temporal ramps elicit a bias in the first head-sweep acceptance rates. (C, D) $^{**}p < 0.01$, $^{***}p < 0.001$. Data show mean and error bars show SEM. (C) Circles indicate mean of individual experiments. (D) The first number is the number of accepted first head-sweeps and the number in brackets is the total number of first head-sweeps for the two phases respectively. Exact *p*-values, *t*-values and degrees of freedom can be found in supplementary table 2.

Supplementary Figure 2

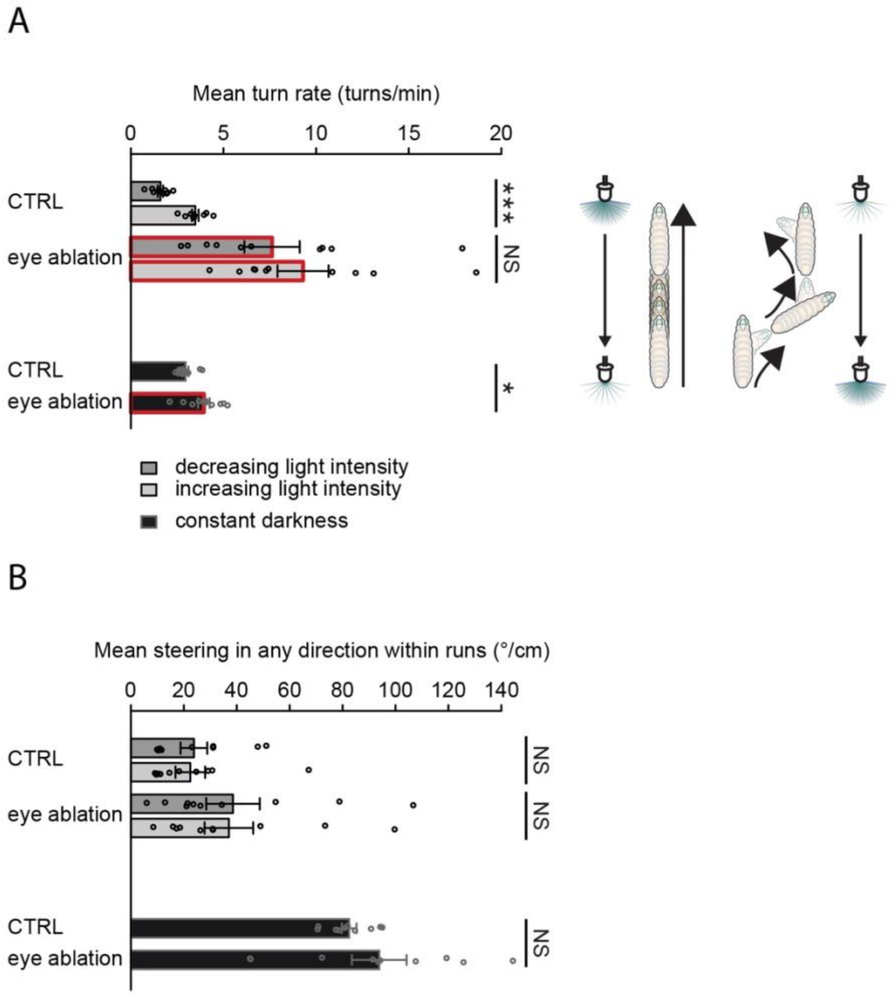

Supplementary Figure 2 Effects of temporal intensity changes on turn frequency and steering within runs.

(A) Bilateral sensing larvae turn more often during the phase of increasing light intensity compared to turn frequency of the decreasing light intensity phase. Unilateral sensing animals do not bias their turn frequency. Unilateral sensing larvae turn more frequently in absence of light. The turn rate was calculated by determining the number of turns per cycle of each intensity change phase and processing these values into turns/min. Afterwards these values were divided by the average number of animals present on the testing plate during the respective cycle. (B) Bi- and unilateral sensing larvae are steering within runs. Both do not bias their steering magnitude in relation to light intensity changes. The steering in any direction within runs was calculated by the delta of the heading direction before the run started and the heading direction after the run stopped. All values were made positive and divided by the run length, to get values of steering in ° per cm. Two- and one-eyed larvae steer more in absence of a light stimulus. (A, B) $*p < 0.05$, $***p < 0.001$, NS = not significant. Data show mean and error bars show SEM. Circles indicate mean of individual experiments. Exact *p*-values, *t*-values and degrees of freedom can be found in supplementary table 2.

Supplementary Figure 3

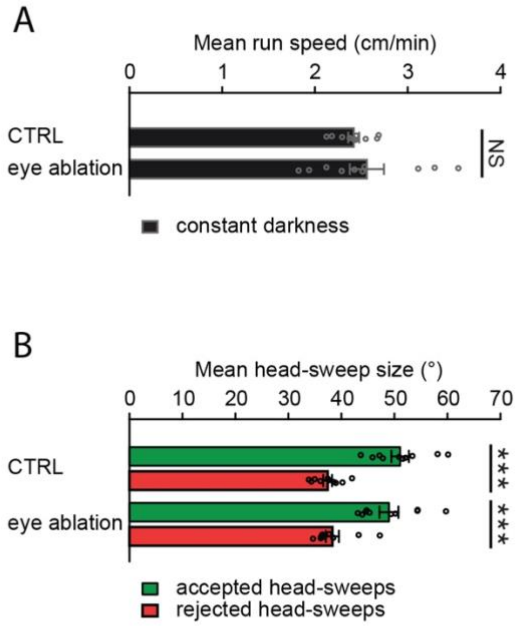

Supplementary Figure 3 Eye ablation has no effects on larval run speed and head-sweep size.

(A) Bi- and unilateral sensing larvae have similar run speeds in absence of a light stimulus. For each run we divided the run length in cm by the run duration in min. We calculated a mean for each experiment (circles) and out of these individual means a final mean of all experiments (bars). (B) Both groups bias their head-sweep size. Accepted head-sweeps are significantly greater than rejected head-sweeps. We calculated the head-sweep size by determining the difference between larval heading direction before a head-sweeps starts and the maximal heading direction (A, B) ***$p < 0.001$, NS = not significant. Data present mean and error bars present SEM. Circles show mean of individual experiments. Exact *p*-values, *t*-values and degrees of freedom can be found in supplementary table 2.

Supplementary Figure 4

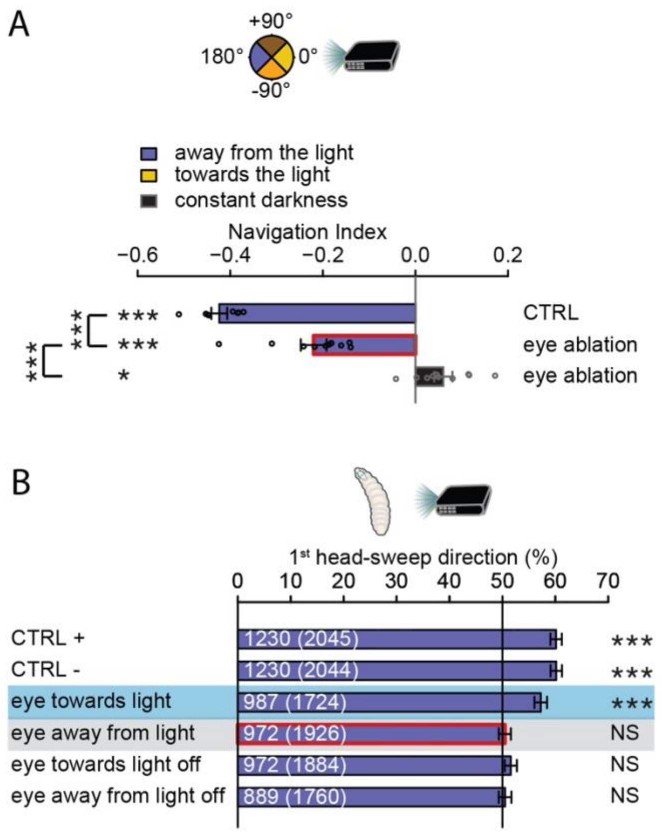

Supplementary Figure 4 Navigation index and direction of all first head-sweeps of bi- and unilateral sensing larvae.

(A) We determined a navigation index, which is defined by the average run velocity towards the light source divided by the average speed irrespective of run direction. The presence of only one eye is sufficient for visual navigation although unilateral sensing larvae navigate significantly worse than control animals. (B) Control larvae bias the direction of their first head-sweeps away from the light source, independent of the light source being present on their right or left side. Unilateral sensing animals are only biasing the direction of their first head-sweeps away from the light, in case their functional eye is on the body-side facing the light source. These animals do not bias the direction of their first head-sweep in any direction under constant darkness conditions. (A, B) *$p < 0.05$, ***$p < 0.001$, NS = not significant. Data present mean and error bars present SEM. (A) Circles show mean of individual experiments. (B) The first number is the number of first head-sweeps directed away from the light source and the number in brackets is the total number of all first head-sweeps. Exact *p*-values, *t*-values and degrees of freedom can be found in supplementary table 2.

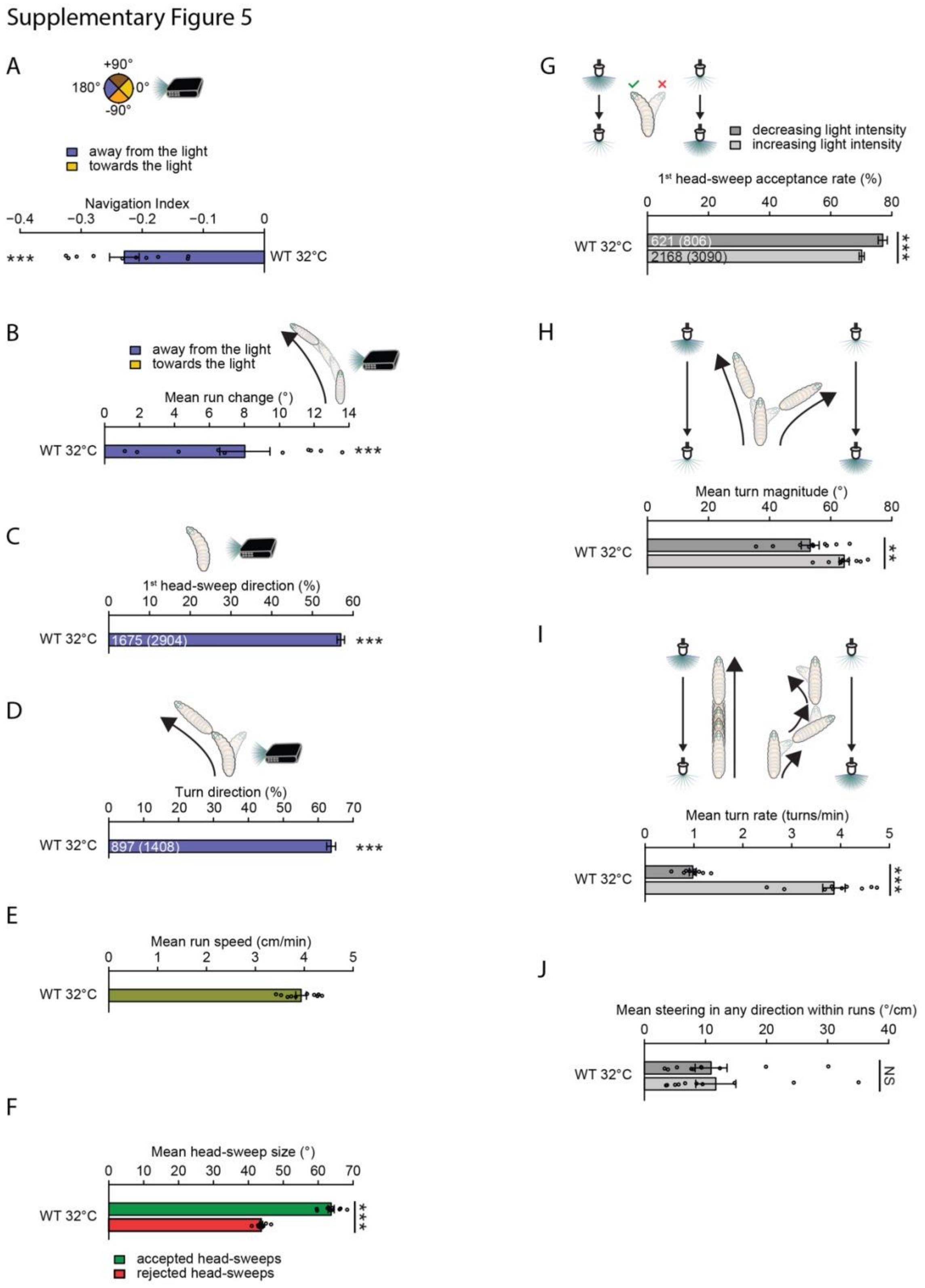

Supplementary Figure 5 Temperature modulates behavioral parameters.

Independent of all modulations the behavioral biases were still be observed. At 32°C larvae are still navigating away from the light source. (B) Larvae are steering within runs away from the light source. (C) They are also biasing the direction of their first head-sweeps and (D) turns away from the light source. We observed less first head-

sweeps and turns in the directional assay. (E) Larval run speed increases to 3.9 cm/min at 32°C. (F) Larvae are still making significantly greater accepted head-sweeps compared to the size of rejected head-sweeps. (G) In the temporal light gradient assay, larvae are also accepting more first head-sweeps in the time phase of decreasing light intensity. Larvae are accepting with a higher probability their first head-sweeps. (H) In the phase of increasing light intensity larvae are making significantly greater turns. (I) The turn frequency is significantly higher in the phase of light intensity increase. (J) At 32°C larvae are still not biasing the magnitude of their steering within runs in relation to light intensity in- or decrease. (A-J) ***p* < 0.01, ****p* < 0.001, NS = not significant. Data present mean and error bars present SEM. (A, B, E, F, H-J) Circles indicate mean of individual experiments. (C) The first number indicates the number of first head-sweeps directed away from the light source and the number in brackets indicates the total number of all first head-sweeps. (D) Turns directed away from the light source are displayed as the first number. The number in brackets displays all the turns. (G) The first numbers are the number of accepted first head-sweeps and the numbers in brackets are the numbers of all first head-sweeps with respect to the two different phases. The exact *p*-values, *t*-values and degrees of freedom can be found in supplementary table 2.

Supplementary Figure 6

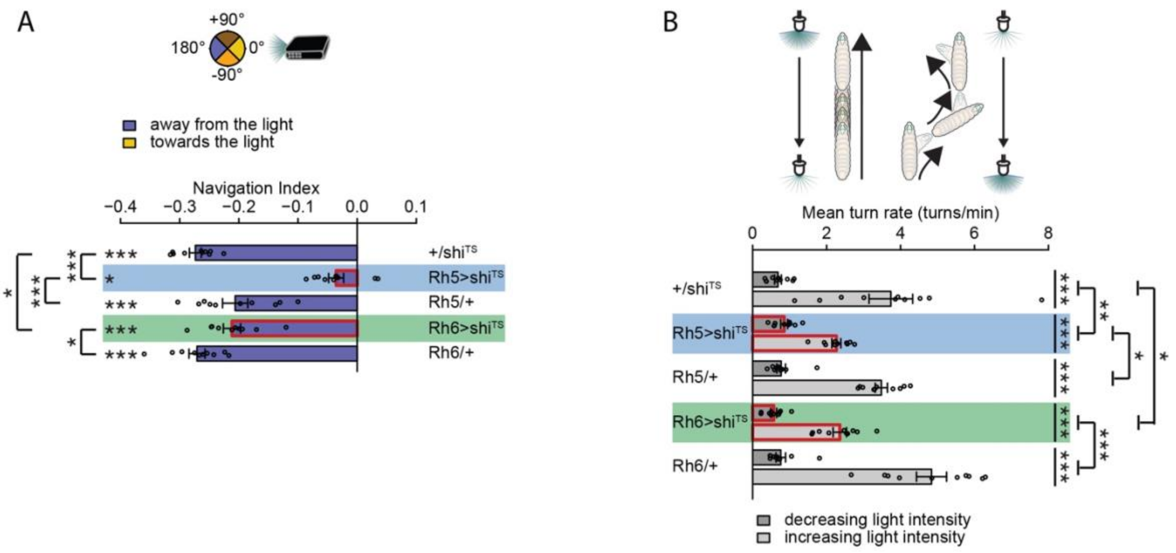

Supplementary Figure 6 Silencing either PR-subtype lead to a decreased navigation index and turn frequency bias.

(A) Larvae lacking either functional Rh5- or Rh6-PRs are able to navigate away from the light source. The navigation indices are decreased in comparison to both controls. (B) Larvae are biasing their turn frequency in dependence of light intensity changes. Larvae with silenced Rh5- or Rh6-PRs have a decreased turn rate bias in comparison to both controls. (A, B) **p* < 0.05, ***p* < 0.01, ****p* < 0.001. Data show mean and error bars show SEM. Circles show mean of individual experiments. For exact *p*-values, *t*-values and degrees of freedom please see supplementary table 2.

Supplementary Figure 7

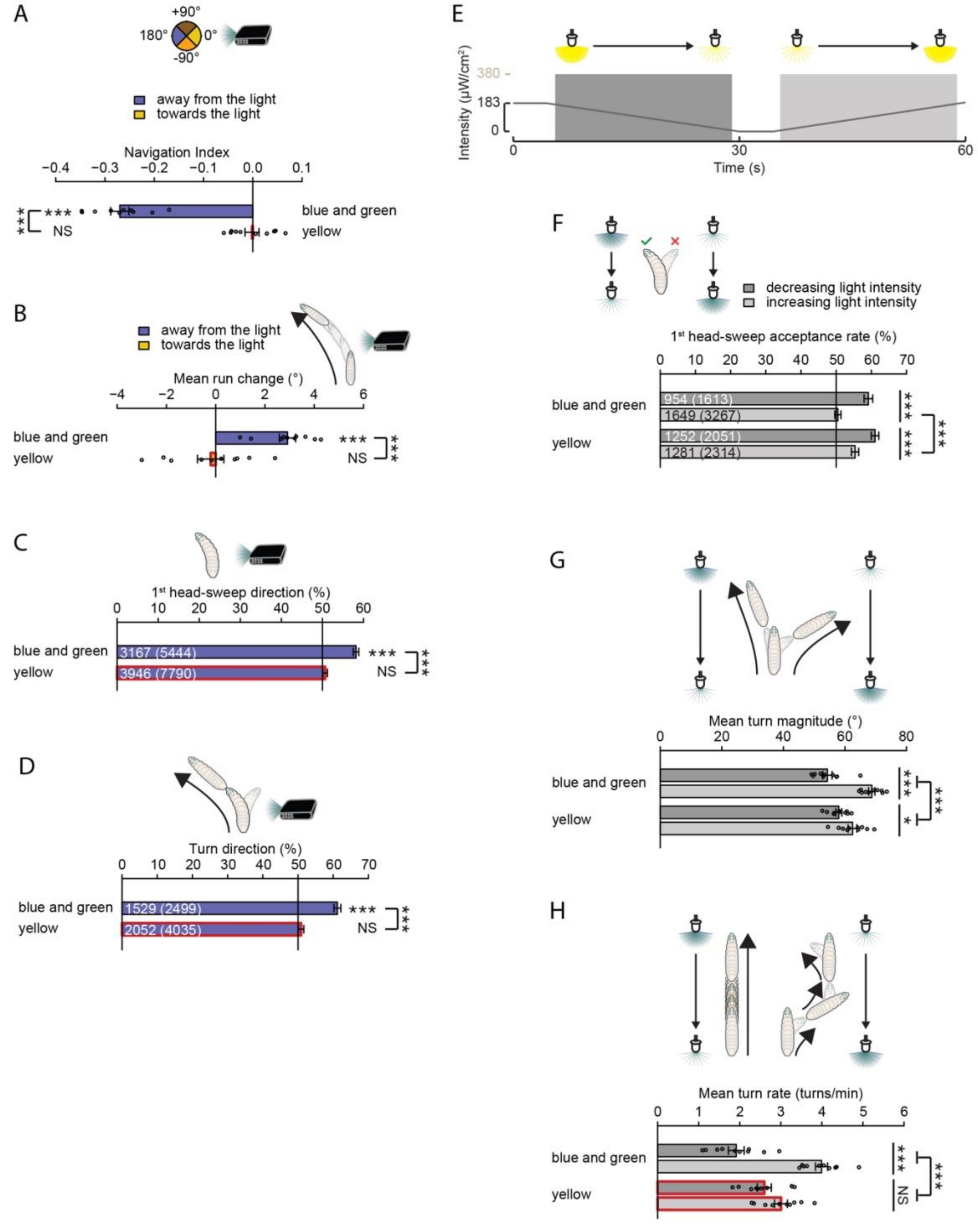

Supplementary Figure 7 Yellow LEDs elicit two behavioral biases mediated by temporal intensity processing.

Larvae were stimulated with energy-equal light emitted by yellow LEDs and by blue and green LEDs. (A) Larvae navigate away from blue and green LEDs, but not from yellow LEDs. (B) Animals steer within runs away from blue and green LEDs, but not from yellow LEDs. (C, D) Larvae bias the direction of their first head-sweeps and of their turns away from blue and green light, but not away from yellow light. (E) In the

temporal light intensity gradient assay, for 25.5 s the light intensity was decreasing linearly from 183 $\mu W/cm^2$ to 0 $\mu W/cm^2$ and increasing linearly with the same speed. The two different phases were spaced by 4.5 s of constant high or low light intensity. One cycle lasted 1 min and was repeated 10-times. For analysis, we were not taken into account the phases of constant light intensity +/- 1 s. (F) Both lighting conditions elicit a bias in the first head-sweep acceptance rates. (G) In both light-scapes larvae make greater turns when light intensity was increasing. (H) Larvae are biasing the turn frequency only in the experiments using the blue and green LEDs. (A-D, F-H) $^*p < 0.05$, $^{***}p < 0.001$, NS = not significant. Data show mean and error bars show SEM. For exact *p*-values, *t*-values and degrees of freedom please see supplementary table 2. (A, B, G, H) Circles show mean of individual experiments. (C) First numbers are the number of first head-sweeps directed away from the light source. Numbers in brackets are the number of all first head-sweeps. (D) First numbers are the number of turns directed away from the light source and the numbers in brackets are the number of all turns. (F) The first numbers indicate accepted first head-sweeps and the numbers in brackets indicate number of all first head-sweeps.

Supplementary Figure 8

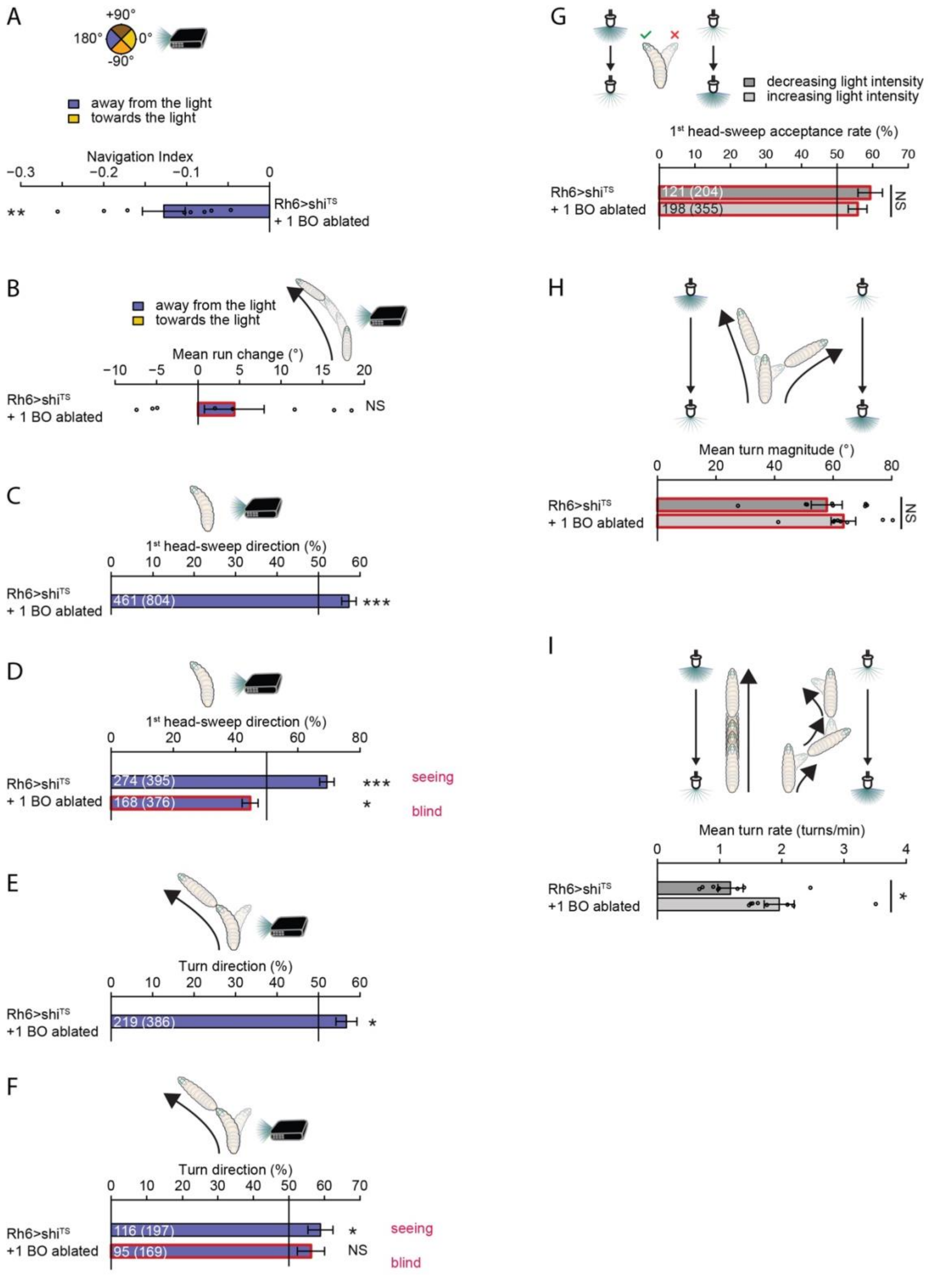

Supplementary Figure 8 Combination of silencing Rh6-PRs and unilateral eye ablation lead to several behavioral defects.

We combined genetically silencing Rh6-PRs and unilateral eye ablation in larvae. (A) These animals are still navigating away from the light source. (B) They are not able

to steer within runs away from the light source. (C) Their first head-sweep direction is biased away from the light source. (D) These animals are just able to bias their first head-sweep direction away from the light source in case their remaining Rh5-PRs were on the body-side facing towards the light source. In one of these experiments, we did not distinguish between larvae lacking the left or the right eye. Therefore, numbers of first head-sweeps and turns do not fit exactly between panels. (E) Unilateral sensing larvae with not functional Rh6-PRs are able to bias their turn direction away from the light. (F) These animals were biasing their turn direction only in case the remaining Rh5-PRs are on the side towards the light source. (G) These animals are not biasing their first head-sweep acceptance rate in relation to light intensity changes. (H) These larvae are not showing a bias of their turn size in dependence of the light intensity changes. (I) These larvae are making significantly more turns in the phase of light intensity increase. (A-I) *$p < 0.05$, **$p < 0.01$, ***$p < 0.001$, NS = not significant. Data represent mean and error bars represent SEM. For exact p-values, t-values and degrees of freedom please see supplementary table 2. (A, B, H, I) Circles are means of individual experiments. (C, D) First numbers indicate the number of first head-sweeps directed away from the light source. Numbers in brackets indicate the number of all first head-sweeps. (E, F) First numbers show the number of turns directed away from the light source and the numbers in brackets show the number of all turns. (G) The first numbers are accepted first head-sweeps and the numbers in brackets are number of all first head-sweeps with respect to the different phases.

Supplementary Figure 9

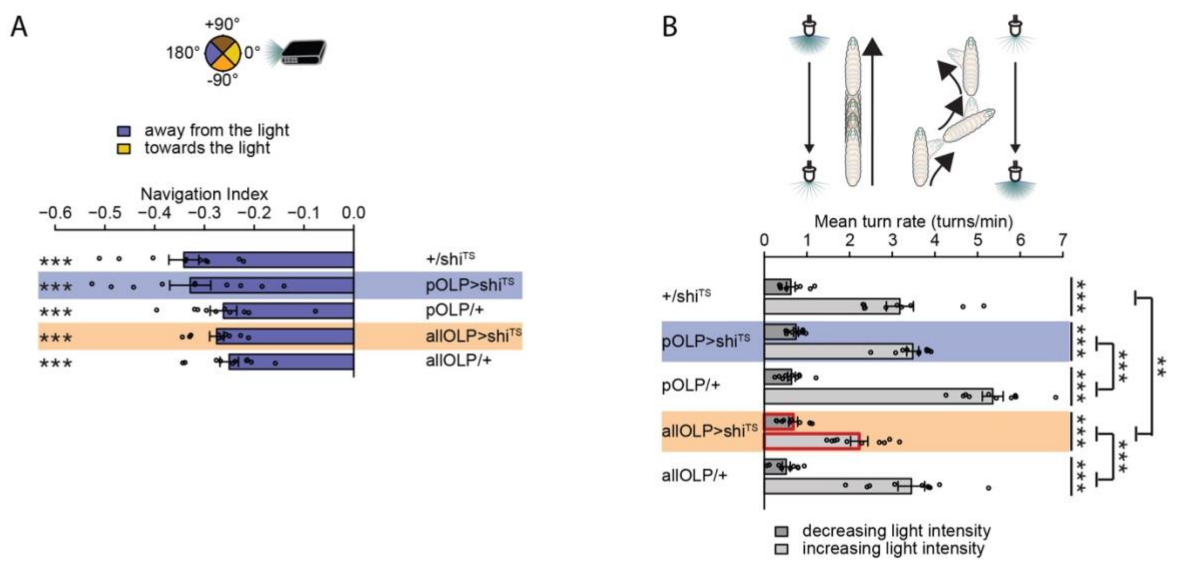

Supplementary Figure 9 Navigation indices and turn frequency biases of larvae lacking either functional pOLP or all OLPs.

(A) The navigation indices of larvae lacking either pOLP or all OLPs differ not from the indices of control animals. (B) Larvae of both genotypes are able to bias their turn frequency with respect to light intensity changes. Larvae lacking functional OLPs possess a decreased turn rate bias in comparison with their respective controls. (A, B) **$p < 0.01$, ***$p < 0.001$. Data show mean and error bars show SEM. For exact p-

values, *t*-values and degrees of freedom please see supplementary table 2. Circles indicate the means of individual experiments.

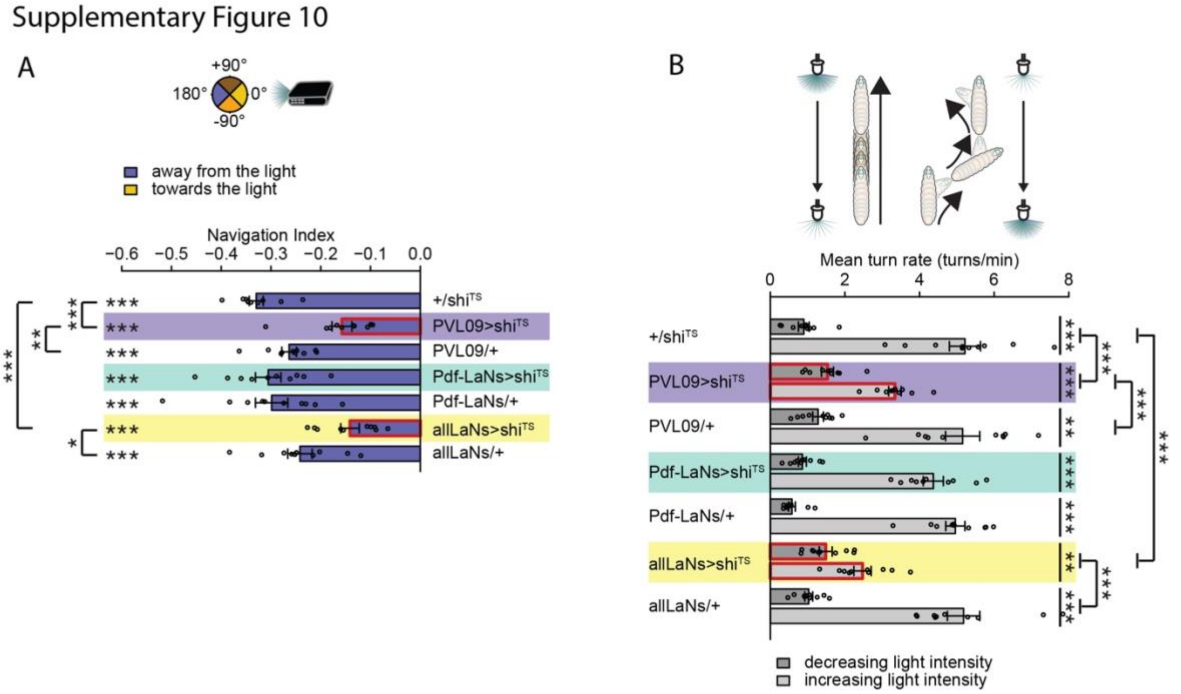

Supplementary Figure 10 Navigation indices and turn frequency biases of larvae lacking either functional PVL09, Pdf-LaNs or all LaNs.

(A) The navigation indices of larvae lacking either PVL09 or all LaNs differ significantly from the indices of the respective controls. (B) Larvae of all genotypes are able to bias their turn rate in relation to temporal light intensity changes. Animals with not functional PVL09 or all LaNs show a decreased turn frequency bias in comparison with the respective controls. (A, B) *$p < 0.01$, **$p < 0.01$, ***$p < 0.001$. Data indicate mean and error bars indicate SEM. For exact *p*-values, *t*-values and degrees of freedom please see supplementary table 2. Circles show the means of individual experiments.

Supplementary Table 1. Results of statistical analysis for main data

Figure	Test used	tested genotype(s)	n	Column1	*p* -value	df	F- or t-value or OR
1D	Fisher's exact test	CTRL (temporal assay)	4798	1. head-sweeps	$1.67 * 10^{-12}$		1.56
1D	Fisher's exact test	eye ablation (temporal assay)	2740	1. head-sweeps	$2.83 * 10^{-9}$		1.59
1D	Fisher's exact test	CTRL (dark) vs eye ablation (dark)	11426	1. head-sweeps	$3.59 * 10^{-10}$		0.78
1F	Two sample t test	CTRL (temporal assay)	10	experiments	$3.36 * 10^{-6}$	18	6.13
1F	Two sample t test	eye ablation (temporal assay)	10	experiments	$3.36 * 10^{-6}$	18	6.60
1F	Two sample t test	CTRL (dark) vs eye ablation (dark)	20	experiments	0.024	18	0.04
2A	Exact binomial test	CTRL	566	turns	0.2118		
2A	Exact binomial test	eye ablation	745	turns	0.0079		
2A	Exact binomial test	eye ablation (dark)	1000	turns	0.548		
2B	Exact binomial test	CTRL	527	1. head-sweeps	0.6495		
2B	Exact binomial test	eye ablation	760	1. head-sweeps	0.0156		
2B	Exact binomial test	eye ablation (dark)	979	1. head-sweeps	1		
2C	One sample t test	CTRL	8	experiments	0.5139	7	1.52
2C	One sample t test	eye ablation	10	experiments	0.8865	9	0.56
2C	One sample t test	eye ablation (dark)	10	experiments	0.9516	9	0.06
3B	Exact binomial test	CTRL +90°	766	1. head-sweeps	$1.77 * 10^{-4}$		
3B	Exact binomial test	CTRL -90°	846	1. head-sweeps	$2.52 * 10^{-7}$		
3B	Exact binomial test	eye towards light	666	1. head-sweeps	0.0234		
3B	Exact binomial test	eye away from light	765	1. head-sweeps	0.5152		
3B	Exact binomial test	eye towards light off	991	1. head-sweeps	0.1910		

3B	Exact binomial test	eye away from light off	883	1. head-sweeps	0.303		
3C	Exact binomial test	CTRL +90°	856	turns	$1.32 * 10^{-15}$		
3C	Exact binomial test	CTRL -90°	890	turns	$3.59 * 10^{-12}$		
3C	Exact binomial test	eye towards light	747	turns	$1.68 * 10^{-12}$		
3C	Exact binomial test	eye away from light	827	turns	0.7031		
3C	Exact binomial test	eye towards light off	991	turns	0.7031		
3C	Exact binomial test	eye away from light off	859	turns	0.7031		
3D	One sample t test	CTRL +90°	8	experiments	$5.15 * 10^{-4}$	7	5.84
3D	One sample t test	CTRL -90°	8	experiments	$5.15 * 10^{-4}$	7	-7.24
3D	One sample t test	eye towards light	10	experiments	0.0442	9	2.76
3D	One sample t test	eye away from light	10	experiments	0.2292	9	1.56
3D	One sample t test	eye towards light off	10	experiments	0.6494	9	-0.64
3D	One sample t test	eye away from light off	10	experiments	0.673	9	0.44
4A	One sample t	*+/shi* ^{TS}	10	experiments	0.011	9	3.33
4A	One sample t	*Rh5>shi* ^{TS}	10	experiments	0.1844	9	-1.44
4A	One sample t	*Rh5/+*	10	experiments	0.0015	9	5.45
4A	One sample t	*Rh6>shi* ^{TS}	10	experiments	0.0078	9	3.73
4A	One sample t	*Rh6/+*	10	experiments	0.0015	9	5.14
4A	One-way ANOVA				3.0 * 10-5	4, 45	8.61
4A	Dunnett's test	*Rh5>shi* ^{TS} vs *+/shi* ^{TS}			0.0122		-3.12
4A	Dunnett's test	*Rh5>shi* ^{TS} vs *Rh5/+*			0.0024		-3.67
4A	Dunnett's test	*Rh6>shi* ^{TS} vs *+/shi* ^{TS}			1		0.05
4A	Dunnett's test	*Rh6>shi* ^{TS} vs *Rh6/+*			0.0412		-2.64

4B	Exact binomial test	*+/shi*^{TS}	1309	1. head-sweeps	$6.3 * 10^{-10}$		
4B	Exact binomial test	*Rh5>shi*^{TS}	1791	1. head-sweeps	0.1857		
4B	Exact binomial test	*Rh5/+*	1523	1. head-sweeps	$2.4 * 10^{-9}$		
4B	Exact binomial test	*Rh6>shi*^{TS}	1457	1. head-sweeps	$3.7 * 10^{-10}$		
4B	Exact binomial test	*Rh6/+*	1941	1. head-sweeps	$6.3 * 10^{-10}$		
4B	Fisher’s exact test	*Rh5>shi*^{TS} vs *+/shi*^{TS}	3100	1. head-sweeps	$3.7 * 10^{-4}$		1.33
4B	Fisher’s exact test	*Rh5>shi*^{TS} vs *Rh5/+*	3314	1. head-sweeps	$9.3 * 10^{-4}$		1.28
4B	Fisher’s exact test	*Rh6>shi*^{TS} vs *+/shi*^{TS}	2766	1. head-sweeps	0.9692		1.01
4B	Fisher’s exact test	*Rh6>shi*^{TS} vs *Rh6/+*	3398	1. head-sweeps	0.5597		0.94
4C	Exact binomial test	*+/shi*^{TS}	560	turns	$6.38 * 10^{-5}$		
4C	Exact binomial test	*Rh5>shi*^{TS}	881	turns	0.0506		
4C	Exact binomial test	*Rh5/+*	646	turns	0.0032		
4C	Exact binomial test	*Rh6>shi*^{TS}	725	turns	0.0032		
4C	Exact binomial test	*Rh6/+*	815	turns	0.0032		
4C	Fisher’s exact test	*Rh5>shi*^{TS} vs *+/shi*^{TS}	1441	turns	0.1182		0.79
4C	Fisher’s exact test	*Rh5>shi*^{TS} vs *Rh5/+*	1527	turns	0.3369		0.89
4C	Fisher’s exact test	*Rh6>shi*^{TS} vs *+/shi*^{TS}	1285	turns	0.3369		0.86
4C	Fisher’s exact test	*Rh6>shi*^{TS} vs *Rh6/+*	1540	turns	0.9182		1.02
4D	Fisher’s exact test	*+/shi*^{TS}	3137	1. head-sweeps	0.0012		1.44
4D	Fisher’s exact test	*Rh5>shi*^{TS}	2207	1. head-sweeps	$1.9 * 10^{-5}$		1.66
4D	Fisher’s exact test	*Rh5/+*	2949	1. head-sweeps	$1.3 * 10^{-4}$		1.56
4D	Fisher’s exact test	*Rh6>shi*^{TS}	1987	1. head-sweeps	0.0513		1.27
4D	Fisher’s exact test	*Rh6/+*	4058	1. head-sweeps	$1.6 * 10^{-13}$		2.16

4D	Fisher's exact test	*Rh5>shiTS vs +/shiTS (decrease)*	1068	1. head-sweeps	0.106		0.77
4D	Fisher's exact test	*Rh5>shiTS vs +/shiTS (increase)*	4276	1. head-sweeps	0.1264		0.89
4D	Fisher's exact test	*Rh5>shiTS vs Rh5/+ (decrease)*	1110	1. head-sweeps	0.8829		0.97
4D	Fisher's exact test	*Rh5>shiTS vs Rh5/+ (increase)*	4046	1. head-sweeps	0.8003		1.03
4D	Fisher's exact test	*Rh6>shiTS vs +/shiTS (decrease)*	869	1. head-sweeps	0.0974		1.35
4D	Fisher's exact test	*Rh6>shiTS vs +/shiTS (increase)*	4255	1. head-sweeps	0.0414		1.19
4D	Fisher's exact test	*Rh6>shiTS vs Rh6/+ (decrease)*	939	1. head-sweeps	0.0414		1.47
4D	Fisher's exact test	*Rh6>shiTS vs Rh6/+ (increase)*	5106	1. head-sweeps	0.0548		0.87
4E	Two sample t test	$+/shi^{TS}$	10	experiments	$8.59 * 10^{-5}$	18	5.46
4E	Two sample t test	$Rh5>shi^{TS}$	10	experiments	$1.42 * 10^{-4}$	18	5.04
4E	Two sample t test	*Rh5/+*	10	experiments	$1.99 * 10^{-4}$	18	4.75
4E	Two sample t test	$Rh6>shi^{TS}$	10	experiments	0.0015	18	3.75
4E	Two sample t test	*Rh6/+*	10	experiments	$4.34 * 10^{-8}$	18	10.02
4E	One-way ANOVA				0.0201	4, 45	3.25
4E	Dunnett's test	$Rh5>shi^{TS}$ vs $+/shi^{TS}$			0.998		-0.22
4E	Dunnett's test	$Rh5>shi^{TS}$ vs *Rh5/+*			0.575		1.23
4E	Dunnett's test	$Rh6>shi^{TS}$ vs $+/shi^{TS}$			0.83		-0.84
4E	Dunnett's test	$Rh6>shi^{TS}$ vs *Rh6/+*			0.03		-2.76
5B	One sample t	$+/\mathrm{shi}^{TS}$	10	experiments	$3.9 * 10^{-5}$	9	7.94
5B	One sample t	$pOLP>shi^{TS}$	10	experiments	0.0043	9	3.94
5B	One sample t	*pOLP/+*	10	experiments	0.0138	9	3.05
5B	One sample t	$allOLP>shi^{TS}$	10	experiments	$1.0 * 10^{-6}$	9	12.94
5B	One sample t	*allOLP/+*	10	experiments	$7.5 * 10^{-8}$	9	18.90

5B	One-way ANOVA				0.455	4, 45	0.93
5C	Exact binomial test	+/shi^{TS}	1625	1. head-sweeps	$1.1 * 10^{-7}$		
5C	Exact binomial test	*pOLP>shi*^{TS}	1388	1. head-sweeps	$2.5 * 10^{-9}$		
5C	Exact binomial test	*pOLP*/+	1127	1. head-sweeps	$5.9 * 10^{-10}$		
5C	Exact binomial test	*allOLP>shi*^{TS}	3746	1. head-sweeps	$8.1 * 10^{-13}$		
5C	Exact binomial test	*allOLP*/+	4009	1. head-sweeps	$9.8 * 10^{-11}$		
5C	Fisher's exact test	*pOLP>shi*^{TS} vs +/*shi*^{TS}	3013	1. head-sweeps	1.75		0.94
5C	Fisher's exact test	*pOLP>shi*^{TS} vs *pOLP*/+	2515	1. head-sweeps	0.687		1.05
5C	Fisher's exact test	*allOLP>shi*^{TS} vs +/*shi*^{TS}	5371	1. head-sweeps	0.697		1.02
5C	Fisher's exact test	*allOLP>shi*^{TS} vs *allOLP* /+	7755	1. head-sweeps	0.957		0.97
5D	Exact binomial test	+/shi^{TS}	674	turns	$5.05 * 10^{-10}$		
5D	Exact binomial test	*pOLP>shi*^{TS}	487	turns	$1.60 * 10^{-5}$		
5D	Exact binomial test	*pOLP*/+	485	turns	$1.60 * 10^{-5}$		
5D	Exact binomial test	*allOLP>shi*^{TS}	1451	turns	$1.60 * 10^{-5}$		
5D	Exact binomial test	*allOLP*/+	1622	turns	$4.12 * 10^{-5}$		
5D	Fisher's exact test	*pOLP>shi*^{TS} vs +/*shi*^{TS}	1161	turns	0.786		0.90
5D	Fisher's exact test	*pOLP>shi*^{TS} vs *pOLP*/+	972	turns	1		1.00
5D	Fisher's exact test	*allOLP>shi*^{TS} vs +/*shi*^{TS}	2125	turns	0.0184		0.76
5D	Fisher's exact test	*allOLP>shi*^{TS} vs *allOLP* /+	3073	turns	0.9189		1.03
5E	Fisher's exact test	+/shi^{TS}	2386	1. head-sweeps	0.0078		1.46
5E	Fisher's exact test	*pOLP>shi*^{TS}	3163	1. head-sweeps	$9.9 * 10^{-4}$		1.48
5E	Fisher's exact test	*pOLP*/+	4241	1. head-sweeps	$9.9 * 10^{-4}$		1.54

5E	Fisher's exact test	*allOLP>shi* ^{TS}	2211	1. head-sweeps	0.8308		1.03
5E	Fisher's exact test	*allOLP* /+	2642	1. head-sweeps	0.0349		1.31
5E	Fisher's exact test	*pOLP>shiTS vs +/shiTS (decrease)*	908	1. head-sweeps	0.8311		0.91
5E	Fisher's exact test	*pOLP>shiTS vs +/shiTS (increase)*	4641	1. head-sweeps	0.5104		0.93
5E	Fisher's exact test	*pOLP>shiTS vs pOLP /+ (decrease)*	999	1. head-sweeps	1		1.00
5E	Fisher's exact test	*pOLP>shiTS vs pOLP/+ (increase)*	6405	1. head-sweeps	0.7208		0.96
5E	Fisher's exact test	*allOLP>shiTS vs +/shiTS (decrease)*	867	1. head-sweeps	0.002		1.72
5E	Fisher's exact test	*allOLP>shiTS vs +/shiTS (increase)*	3730	1. head-sweeps	0.0179		1.21
5E	Fisher's exact test	*allOLP>shiTS vs allOLP /+ (decrease)*	841	1. head-sweeps	0.8129		0.94
5E	Fisher's exact test	*allOLP>shiTS vs allOLP/+ (increase)*	4012	1. head-sweeps	$4.7 * 10^{-5}$		0.74
5F	Two sample t test	+/shi^{TS}	10	experiments	$2.86 * 10^{-6}$	18	7.16
5F	Two sample t test	*pOLP>shi* ^{TS}	10	experiments	$1.03 * 10^{-5}$	18	6.16
5F	Two sample t test	*pOLP* /+	10	experiments	$6.10 * 10^{-7}$	18	8.40
5F	Two sample t test	*allOLP>shi* ^{TS}	10	experiments	$1.60 * 10^{-4}$	18	4.75
5F	Two sample t test	*allOLP* /+	10	experiments	$9.27 * 10^{-6}$	18	6.35
5F	One-way ANOVA				0.818	4, 45	0.39
6A	One sample t	*+/shi* ^{TS}	10	experiments	$2.2 * 10^{-4}$	9	6.05
6A	One sample t	*PVL09>shi* ^{TS}	10	experiments	0.0094	9	3.29
6A	One sample t	*PVL09* /+	10	experiments	$9.0 * 10^{-5}$	9	7.19
6A	One sample t	*Pdf-LaNs>shi* ^{TS}	10	experiments	$6.5 * 10^{-6}$	9	11.74
6A	One sample t	*Pdf-LaNs* /+	10	experiments	$8.0 * 10^{-5}$	9	7.57
6A	One sample t	*allLaNs>shi* ^{TS}	10	experiments	$1.8 * 10^{-4}$	9	6.39
6A	One sample t	*allLaNs* /+	10	experiments	$1.7 * 10^{-5}$	9	9.63

6A	One-way ANOVA				$8.7 * 10^{-7}$	6, 63	8.53
6A	Dunnett's test	*PVL09>shi*^{TS} *vs* +/*shi*^{TS}			<0.001		-4.45
6A	Dunnett's test	*PVL09>shiTS vs PVL09/+*			0.1825		-2.12
6A	Dunnett's test	*Pdf-LaNs>shi*^{TS} *vs* +/*shi*^{TS}			0.9821		0.60
6A	Dunnett's test	*Pdf-LaNs>shi*^{TS} *vs Pdf-LaNs/+*			0.9973		-0.41
6A	Dunnett's test	*allLaNs>shi*^{TS} *vs* +/*shi*^{TS}			0.0418		-2.75
6A	Dunnett's test	*allLaNs>shi*^{TS} *vs allLaNs/+*			0.026		-2.93
6B	Exact binomial test	+/*shi*^{TS}	2130	1. head-sweeps	$7.3 * 10^{-8}$		
6B	Exact binomial test	*PVL09>shi*^{TS}	1740	1. head-sweeps	0.0104		
6B	Exact binomial test	*PVL09*/+	3230	1. head-sweeps	$5.9 * 10^{-13}$		
6B	Exact binomial test	*Pdf-LaNs>shi*^{TS}	2263	1. head-sweeps	$5.9 * 10^{-13}$		
6B	Exact binomial test	*Pdf-LaNs* /+	1582	1. head-sweeps	$5.7 * 10^{-9}$		
6B	Exact binomial test	*allLaNs>shi*^{TS}	1841	1. head-sweeps	0.0622		
6B	Exact binomial test	*allLaNs* /+	1211	1. head-sweeps	$3.7 * 10^{-10}$		
6B	Fisher's exact test	*PVL09>shi*^{TS} *vs* +/*shi*^{TS}	3870	1. head-sweeps	0.1372		1.12
6B	Fisher's exact test	*PVL09>shiTS vs PVL09/+*	4970	1. head-sweeps	0.0498		1.14
6B	Fisher's exact test	*Pdf-LaNs>shi*^{TS} *vs* +/*shi*^{TS}	4393	1. head-sweeps	0.2675		0.93
6B	Fisher's exact test	*Pdf-LaNs>shi*^{TS} *vs Pdf-LaNs/+*	3845	1. head-sweeps	0.8683		0.99
6B	Fisher's exact test	*allLaNs>shi*^{TS} *vs* +/*shi*^{TS}	3971	1. head-sweeps	0.0498		1.16
6B	Fisher's exact test	*allLaNs>shi*^{TS} *vs allLaNs/+*	3052	1. head-sweeps	$8.9 * 10^{-4}$		1.33
6C	Exact binomial test	+/*shi*^{TS}	702	turns	$3.32 * 10^{-12}$		
6C	Exact binomial test	*PVL09>shi*^{TS}	843	turns	0.0084		
6C	Exact binomial test	*PVL09*/+	1366	turns	$3.32 * 10^{-12}$		
6C	Exact binomial test	*Pdf-LaNs>shi*^{TS}	900	turns	$6.38 * 10^{-11}$		

6C	Exact binomial test	*Pdf-LaNs /+*	606	turns	1.23 * 10^{-9}		
6C	Exact binomial test	*allLaNs>shi*^{TS}	915	turns	0.0551		
6C	Exact binomial test	*allLaNs /+*	515	turns	1.42 * 10^{-4}		
6C	Fisher’s exact test	*PVL09>shi*^{TS} *vs +/shi*^{TS}	1545	turns	0.0027		0.71
6C	Fisher’s exact test	*PVL09>shiTS vs PVL09/+*	2209	turns	0.0474		0.82
6C	Fisher’s exact test	*Pdf-LaNs>shi*^{TS} *vs +/shi*^{TS}	1602	turns	0.5239		0.92
6C	Fisher’s exact test	*Pdf-LaNs>shi*^{TS} *vs Pdf-LaNs/+*	1506	turns	0.5892		0.94
6C	Fisher’s exact test	*allLaNs>shi*^{TS} *vs +/shi*^{TS}	1617	turns	4.48 * 10^{-4}		0.67
6C	Fisher’s exact test	*allLaNs>shi*^{TS} *vs allLaNs/+*	1430	turns	0.0788		0.80
6D	Fisher’s exact test	*+/shi*^{TS}	4260	1. head-sweeps	1.5 * 10^{-4}		1.45
6D	Fisher’s exact test	*PVL09>shi*^{TS}	3463	1. head-sweeps	0.9368		1.01
6D	Fisher’s exact test	*PVL09/+*	4604	1. head-sweeps	3.0 * 10^{-5}		1.41
6D	Fisher’s exact test	*Pdf-LaNs>shi*^{TS}	4263	1. head-sweeps	2.7 * 10^{-5}		1.52
6D	Fisher’s exact test	*Pdf-LaNs /+*	4107	1. head-sweeps	1.9 * 10^{-4}		1.52
6D	Fisher’s exact test	*allLaNs>shi*^{TS}	2788	1. head-sweeps	0.7371		1.04
6D	Fisher’s exact test	*allLaNs /+*	4420	1. head-sweeps	6.2 * 10^{-8}		1.69
6D	Fisher’s exact test	*PVL09>shiTS vs +/shiTS (decrease)*	1688	1. head-sweeps	0.1477		1.21
6D	Fisher’s exact test	*PVL09>shiTS vs +/shiTS (increase)*	6035	1. head-sweeps	0.0041		0.84
6D	Fisher’s exact test	*PVL09>shiTS vs PVL09/+ (decrease)*	1982	1. head-sweeps	0.9226		1.02
6D	Fisher’s exact test	*PVL09>shiTS vs PVL09/+ (increase)*	6085	1. head-sweeps	2.7 * 10^{-7}		0.73
6D	Fisher’s exact test	*Pdf-LaNs>shiTS vs +/shiTS (decrease)*	1303	1. head-sweeps	0.4548		0.90
6D	Fisher’s exact test	*Pdf-LaNs>shiTS vs +/shiTS (increase)*	7220	1. head-sweeps	0.2428		0.94
6D	Fisher’s exact test	*Pdf-LaNs>shiTS vs Pdf-LaNs/+ (decrease)*	1110	1. head-sweeps	0.1063		0.76
6D	Fisher’s exact test	*Pdf-LaNs>shiTS vs Pdf-LaNs/+ (increase)*	7260	1. head-sweeps	2.7 * 10^{-7}		0.77
6D	Fisher’s exact test	*allLaNs>shiTS vs +/shiTS (decrease)*	1664	1. head-sweeps	1		1.00

6D	Fisher's exact test	*allLaNs>shiTS vs +/shiTS (increase)*	5384	1. head-sweeps	$5.8 * 10^{-7}$		0.72
6D	Fisher's exact test	*allLaNs>shiTS vs allLaNs/+ (decrease)*	1772	1. head-sweeps	0.1477		1.21
6D	Fisher's exact test	*allLaNs>shiTS vs allLaNs/+ (increase)*	5436	1. head-sweeps	$1.5 * 10^{-5}$		0.75
6E	Two sample t test	*+/shi* ^{TS}	10	experiments	$3.84 * 10^{-8}$	18	9.87
6E	Two sample t test	*PVL09>shi* ^{TS}	10	experiments	$1.24 * 10^{-4}$	18	5.13
6E	Two sample t test	*PVL09/+*	10	experiments	0.0022	18	3.56
6E	Two sample t test	*Pdf-LaNs>shi* ^{TS}	10	experiments	$1.35 * 10^{-4}$	18	5.22
6E	Two sample t test	*Pdf-LaNs /+*	10	experiments	$1.50 * 10^{-4}$	18	4.94
6E	Two sample t test	*allLaNs>shi* ^{TS}	10	experiments	0.0015	18	3.81
6E	Two sample t test	*allLaNs /+*	10	experiments	$1.23 * 10^{-9}$	18	12.80
6E	One-way ANOVA				$5.4 * 10^{-7}$	6, 63	8.84
6E	Dunnett's test	*PVL09>shi* ^{TS} *vs +/shi* ^{TS}			0.0024		-3.72
6E	Dunnett's test	*PVL09>shiTS vs PVL09/+*			0.7852		-1.11
6E	Dunnett's test	*Pdf-LaNs>shi* ^{TS} *vs +/shi* ^{TS}			0.5408		-1.47
6E	Dunnett's test	*Pdf-LaNs>shi* ^{TS} *vs Pdf-LaNs/+*			0.6846		-1.26
6E	Dunnett's test	*allLaNs>shi* ^{TS} *vs +/shi* ^{TS}			0.0016		-3.85
6E	Dunnett's test	*allLaNs>shi* ^{TS} *vs allLaNs/+*			< 0.001		-5.66

Supplementary Table 2. Results of statistical analysis for supplementary data

Sup-Figure	Test used	tested genotype(s)	n	Column1	*p* -value	df	F- or t-value or OR
1C	Two sample t test	WT (50ms)	10	experiments	$1.21 * 10^{-4}$	18	5.20
1C	Two sample t test	WT (200ms)	10	experiments	0.001	18	3.91
1C	Two sample t test	WT (50ms) vs WT (200ms)	20	experiments	0.2306	18	1.24
1D	Fisher's exact test	WT (50ms)	4540	1. head-sweeps	$2.55 * 10^{-38}$		2.36
1D	Fisher's exact test	WT (200ms)	5147	1. head-sweeps	$6.91 * 10^{-9}$		1.39
1D	Fisher's exact test	WT (50ms) vs WT (200ms) (decrease)	3501	1. head-sweeps	0.007551		1.21
1D	Fisher's exact test	WT (50ms) vs WT (200ms) (increase)	6186	1. head-sweeps	$6.61 * 10^{-11}$		0.72
2A	Two sample t test	CTRL (temporal assay)	10	experiments	$3.52 * 10^{-7}$	18	8.19
2A	Two sample t test	eye ablation (temporal assay)	10	experiments	0.421	18	0.82
2A	Two sample t test	CTRL (dark) vs eye ablation (dark)	20	experiments	0.0113	18	-2.82
2B	Two sample t test	CTRL (temporal assay)	10	experiments	0.9113	18	-0.18
2B	Two sample t test	eye ablation (temporal assay)	10	experiments	0.9113	18	-0.11
2B	Two sample t test	CTRL (dark) vs eye ablation (dark)	20	experiments	0.3023	18	-1.06
3A	Two sample t test	CTRL (dark) vs eye ablation (dark)	10	experiments	0.4726	18	0.73
3B	Two sample t test	CTRL (dark)	10	experiments	$1.5 * 10^{-6}$	18	7.38
3B	Two sample t test	eye ablation (dark)	10	experiments	$1.11 * 10^{-4}$	18	4.92
3B	Two sample t test	CTRL (dark) vs eye ablation (dark)	20	experiments	0.0812	18	-1.85

4A	One sample t test	CTRL	8	experiments	$1.37 * 10^{-7}$	7	-24.68
4A	One sample t test	eye ablation	10	experiments	$3.67 * 10^{-5}$	9	-7.90
4A	One sample t test	eye ablation (dark)	10	experiments	0.0135	9	3.06
4A	Two sample t test	CTRL vs eye ablation	18	experiments	$3.32 * 10^{-7}$	16	5.86
4A	Two sample t test	eye ablation vs eye ablation (dark)	20	experiments	$2.44 * 10^{-5}$	18	-8.22
4B	Exact binomial test	CTRL +90°	2045	1. head-sweeps	$6.6 * 10^{-16}$		
4B	Exact binomial test	CTRL -90°	2044	1. head-sweeps	$6.6 * 10^{-16}$		
4B	Exact binomial test	eye towards light	1724	1. head-sweeps	$3.78 * 10^{-9}$		
4B	Exact binomial test	eye away from light	1926	1. head-sweeps	0.6985		
4B	Exact binomial test	eye towards light off	1884	1. head-sweeps	0.261		
4B	Exact binomial test	eye away from light off	1760	1. head-sweeps	0.6985		
5A	One sample t test	WT 32°C	10	experiments	$5.6 * 10^{-6}$	9	-9.47
5B	One sample t test	WT 32°C	10	experiments	$3.39 * 10^{-4}$	9	5.59
5C	Exact binomial test	WT 32°C	2940	1. head-sweeps	$4.18 * 10^{-14}$		
5D	Exact binomial test	WT 32°C	1408	turns	$2.2 * 10^{-16}$		
5F	Two sample t test	WT 32°C	10	experiments	$8.19 * 10^{-14}$	18	20.19
5G	Fisher’s exact test	WT 32°C	3896	1. head-sweeps	$9.44 * 10^{-5}$		1.43

5H	Two sample t test	WT 32°C	10	experiments	0.0039	18	3.32
5I	Two sample t test	WT 32°C	10	experiments	$4.91 * 10^{-10}$	18	12.02
5J	Two sample t test	WT 32°C	10	experiments	0.8567	18	0.18
6A	One sample t test	*+/shi* ^{TS}	10	experiments	$2.86 * 10^{-9}$	9	-27.32
6A	One sample t test	*Rh5>shi* ^{TS}	10	experiments	0.0208	9	-2.80
6A	One sample t test	*Rh5/+*	10	experiments	$6.18 * 10^{-6}$	9	-9.62
6A	One sample t test	*Rh6>shi* ^{TS}	10	experiments	$2.55 * 10^{-7}$	9	-14.48
6A	One sample t test	*Rh6/+*	10	experiments	$2.41 * 10^{-8}$	9	-19.87
6A	One-way ANOVA				$1.22 * 10^{-14}$	4, 45	41.98
6A	Dunnett's test	*Rh5>shi* ^{TS} vs *+/shi* ^{TS}			<0.001		11.24
6A	Dunnett's test	*Rh5>shi* ^{TS} vs *Rh5/+*			<0.001		8.06
6A	Dunnett's test	*Rh6>shi* ^{TS} vs *+/shi* ^{TS}			0.0199		2.93
6A	Dunnett's test	*Rh6>shi* ^{TS} vs *Rh6/+*			0.0279		2.79
6B	Two sample t test	*+/shi* ^{TS}	10	experiments	$7.31 * 10^{-5}$	18	5.11
6B	Two sample t test	*Rh5>shi* ^{TS}	10	experiments	$5.19 * 10^{-8}$	18	9.19
6B	Two sample t test	*Rh5/+*	10	experiments	$3.98 * 10^{-10}$	18	13.44
6B	Two sample t test	*Rh6>shi* ^{TS}	10	experiments	$5.19 * 10^{-8}$	18	9.04
6B	Two sample t test	*Rh6/+*	10	experiments	$4.38 * 10^{-8}$	18	9.57
6B	One-way ANOVA				$1.13 * 10^{-6}$	4, 45	11.89
6B	Dunnett's test	*Rh5>shi* ^{TS} vs *+/shi* ^{TS}			0.0017		-3.78
6B	Dunnett's test	*Rh5>shi* ^{TS} vs *Rh5/+*			0.0169		-2.99

6B	Dunnett's test	*Rh6>shi* ^{TS} vs +/*shi* ^{TS}			0.0198		-2.92
6B	Dunnett's test	*Rh6>shi* ^{TS} vs *Rh6*/+			< 0.001		-5.28
7A	One sample t test	blue and green	10	experiments	$2.73 * 10^{-7}$	9	-14.67
7A	One sample t test	yellow	10	experiments	0.9146	9	-0.11
7A	Two sample t test	blue and green vs yellow	20	experiments	$9.75 * 10^{-10}$	18	-11.52
7B	One sample t test	blue and green	10	experiments	$2.0 * 10^{-5}$	9	8.83
7B	One sample t test	yellow	10	experiments	0.7135	9	-0.38
7B	Two sample t test	blue and green vs yellow	20	experiments	$1.02 * 10^{-4}$	18	4.96
7C	Exact binomial test	blue and green	5444	1. head-sweeps	$4.4 * 10^{-16}$		
7C	Exact binomial test	yellow	7790	1. head-sweeps	0.2525		
7C	Fisher's exact test	blue and green vs yellow	13234	1. head-sweeps	$2.2 * 10^{-16}$		1.35
7D	Exact binomial test	blue and green	2499	turns	$4.4 * 10^{-16}$		
7D	Exact binomial test	yellow	4035	turns	0.2844		
7D	Fisher's exact test	blue and green vs yellow	6534	turns	$3.2 * 10^{-16}$		1.52
7F	Fisher's exact test	blue and green	4880	1. head-sweeps	$2.29 * 10^{-8}$		1.42
7F	Fisher's exact test	yellow	4365	1. head-sweeps	$1.57 * 10^{-4}$		1.26
7F	Fisher's exact test	blue and green vs yellow (decrease)	3664	1. head-sweeps	0.2479		0.92
7F	Fisher's exact test	blue and green vs yellow (increase)	5581	1. head-sweeps	$6.58 * 10^{-4}$		0.82

7G	Two sample t test	blue and green	10	experiments	$6.55 * 10^{-7}$	18	7.84
7G	Two sample t test	yellow	10	experiments	0.0193	18	2.57
7G	Two sample t test	blue and green vs yellow	20	experiments	$5.80 * 10^{-5}$	18	-5.22
7H	Two sample t test	blue and green	10	experiments	$1.75 * 10^{-7}$	18	8.59
7H	Two sample t test	yellow	10	experiments	0.097	18	-1.75
7H	Two sample t test	blue and green vs yellow	20	experiments	$6.64 * 10^{-10}$	18	-11.80
8A	One sample t test	*Rh6>shi*^{TS} + 1 BO ablated	8	experiments	0.0017	7	-4.93
8B	One sample t test	*Rh6>shi*^{TS} + 1 BO ablated	8	experiments	0.2647	7	1.21
8C	Exact binomial test	*Rh6>shi*^{TS} + 1 BO ablated	804	1. head-sweeps	$5.37 * 10^{-5}$		
8D	Exact binomial test	*Rh6>shi*^{TS} + 1 BO ablated (seeing)	395	1. head-sweeps	$2.84 * 10^{-14}$		
8D	Exact binomial test	*Rh6>shi*^{TS} + 1 BO ablated (blind)	376	1. head-sweeps	0.0442		
8E	Exact binomial test	*Rh6>shi*^{TS} + 1 BO ablated	386	turns	0.0228		
8F	Exact binomial test	*Rh6>shi*^{TS} + 1 BO ablated (seeing)	197	turns	0.0228		
8F	Exact binomial test	*Rh6>shi*^{TS} + 1 BO ablated (blind)	169	turns	0.1237		
8G	Fisher’s exact test	*Rh6>shi*^{TS} + 1 BO ablated	559	1. head-sweeps	0.4258		1.16
8H	Two sample t test	*Rh6>shi*^{TS} + 1 BO ablated	8	experiments	0.4134	14	0.84

8I	Two sample t test	*Rh6>shi* ^{TS} + 1 BO ablated	8	experiments	0.0265	14	2.48
9A	One sample t	+/shi^{TS}	10	experiments	$2.09 * 10^{-6}$	9	-11.33
9A	One sample t	*pOLP>shi* ^{TS}	10	experiments	$2.26 * 10^{-5}$	9	-7.98
9A	One sample t	*pOLP*/+	10	experiments	$5.28 * 10^{-6}$	9	-9.80
9A	One sample t	*allOLP>shi* ^{TS}	10	experiments	$5.69 * 10^{-8}$	9	-19.49
9A	One sample t	*allOLP*/+	10	experiments	$6.97 * 10^{-7}$	9	-13.51
9A	One-way ANOVA				0.0876	4, 45	2.17
9B	Two sample t test	+/shi^{TS}	10	experiments	$5.43 * 10^{-7}$	18	7.68
9B	Two sample t test	*pOLP>shi* ^{TS}	10	experiments	$1.47 * 10^{-12}$	18	18.00
9B	Two sample t test	*pOLP*/+	10	experiments	$2.52 * 10^{-12}$	18	18.16
9B	Two sample t test	*allOLP>shi* ^{TS}	10	experiments	$2.11 * 10^{-6}$	18	6.84
9B	Two sample t test	*allOLP*/+	10	experiments	$7.76 * 10^{-8}$	18	8.97
9B	One-way ANOVA				$2.54 * 10^{-11}$	4, 45	26.46
9B	Dunnett's test	*pOLP>shi* ^{TS} vs +/*shi* ^{TS}			0.9451		0.58
9B	Dunnett's test	*pOLP>shi* ^{TS} vs *pOLP*/+			< 0.001		-6.27
9B	Dunnett's test	*allOLP>shi* ^{TS} vs +/*shi* ^{TS}			0.0093		-3.20
9B	Dunnett's test	*allOLP>shi* ^{TS} vs *allOLP* /+			< 0.001		-4.40
10A	One sample t	+/*shi* ^{TS}	10	experiments	$1.63 * 10^{-8}$	9	-23.33
10A	One sample t	*PVL09>shi* ^{TS}	10	experiments	$2.98 * 10^{-5}$	9	-7.86
10A	One sample t	*PVL09*/+	10	experiments	$8.37 * 10^{-8}$	9	-17.91
10A	One sample t	*Pdf-LaNs>shi* ^{TS}	10	experiments	$1.89 * 10^{-6}$	9	-11.93

10A	One sample t	*Pdf-LaNs /+*	10	experiments	$1.01 * 10^{-5}$	9	-9.19
10A	One sample t	*allLaNs>shi*^{TS}	10	experiments	$3.34 * 10^{-5}$	9	-7.59
10A	One sample t	*allLaNs /+*	10	experiments	$6.87 * 10^{-6}$	9	-9.89
10A	One-way ANOVA				$2.74 * 10^{-8}$	6, 63	10.90
10A	Dunnett's test	*PVL09>shi*^{TS} *vs +/shi*^{TS}			< 0.001		5.47
10A	Dunnett's test	*PVL09>shiTS vs PVL09/+*			0.0074		3.37
10A	Dunnett's test	*Pdf-LaNs>shi*^{TS} *vs +/shi*^{TS}			0.9468		0.76
10A	Dunnett's test	*Pdf-LaNs>shi*^{TS} *vs Pdf-LaNs/+*			0.9999		-0.22
10A	Dunnett's test	*allLaNs>shi*^{TS} *vs +/shi*^{TS}			< 0.001		5.98
10A	Dunnett's test	*allLaNs>shi*^{TS} *vs allLaNs/+*			0.0121		3.19
10B	Two sample t test	*+/shi*^{TS}	10	experiments	$2.85 * 10^{-8}$	18	9.80
10B	Two sample t test	*PVL09>shi*^{TS}	10	experiments	$4.53 * 10^{-7}$	18	7.74
10B	Two sample t test	*PVL09/+*	10	experiments	$3.03 * 10^{-7}$	18	8.07
10B	Two sample t test	*Pdf-LaNs>shi*^{TS}	10	experiments	$1.54 * 10^{-9}$	18	12.11
10B	Two sample t test	*Pdf-LaNs /+*	10	experiments	$2.86 * 10^{-11}$	18	16.06
10B	Two sample t test	*allLaNs>shi*^{TS}	10	experiments	0.0031	18	3.42
10B	Two sample t test	*allLaNs /+*	10	experiments	$4.89 * 10^{-8}$	18	9.28
10B	One-way ANOVA				$1.41 * 10^{-12}$	6, 63	19.19
10B	Dunnett's test	*PVL09>shi*^{TS} *vs +/shi*^{TS}			<0.001		-5.78
10B	Dunnett's test	*PVL09>shiTS vs PVL09/+*			<0.001		-4.76
10B	Dunnett's test	*Pdf-LaNs>shi*^{TS} *vs +/shi*^{TS}			0.295		-1.86
10B	Dunnett's test	*Pdf-LaNs>shi*^{TS} *vs Pdf-LaNs/+*			0.228		-2.00
10B	Dunnett's test	*allLaNs>shi*^{TS} *vs +/shi*^{TS}			<0.001		-7.67

10B	Dunnett’s test	*allLaNs>shi*^{TS} *vs allLaNs/+*			<0.001		-7.28
